# A machine learning approach to evaluate the impact of virtual balance/cognitive training on fall risk in older women

**DOI:** 10.3389/fncom.2024.1390208

**Published:** 2024-05-14

**Authors:** Beata Sokołowska, Wiktor Świderski, Edyta Smolis-Bąk, Ewa Sokołowska, Teresa Sadura-Sieklucka

**Affiliations:** ^1^Bioinformatics Laboratory, Mossakowski Medical Research Institute, Polish Academy of Sciences, Warsaw, Poland; ^2^Department of Geriatrics, National Institute of Geriatrics, Rheumatology and Rehabilitation, Warsaw, Poland; ^3^Department of Coronary Artery Disease and Cardiac Rehabilitation, National Institute of Cardiology, Warsaw, Poland; ^4^Department of Developmental Psychology, Faculty of Social Sciences, Institute of Psychology, The John Paul II Catholic University of Lublin, Lublin, Poland

**Keywords:** machine learning, *k*-NN algorithm, virtual reality, body balance, postural stability, balance and cognitive training, aging, fall risk

## Abstract

**Introduction:**

Novel technologies based on virtual reality (VR) are creating attractive virtual environments with high ecological value, used both in basic/clinical neuroscience and modern medical practice. The study aimed to evaluate the effects of VR-based training in an elderly population.

**Materials and methods:**

The study included 36 women over the age of 60, who were randomly divided into two groups subjected to balance-strength and balance-cognitive training. The research applied both conventional clinical tests, such as (a) the Timed Up and Go test, (b) the five-times sit-to-stand test, and (c) the posturographic exam with the Romberg test with eyes open and closed. Training in both groups was conducted for 10 sessions and embraced exercises on a bicycle ergometer and exercises using non-immersive VR created by the ActivLife platform. Machine learning methods with a *k*-nearest neighbors classifier, which are very effective and popular, were proposed to statistically evaluate the differences in training effects in the two groups.

**Results and conclusion:**

The study showed that training using VR brought beneficial improvement in clinical tests and changes in the pattern of posturographic trajectories were observed. An important finding of the research was a statistically significant reduction in the risk of falls in the study population. The use of virtual environments in exercise/training has great potential in promoting healthy aging and preventing balance loss and falls among seniors.

## 1 Introduction

We are currently witnessing dynamic changes in the structure of societies around the world (Lutz et al., 2008; Sanderson and Scherbov, 2010; United Nations, 2017). Medical advances, improved living conditions and increased life expectancy are making old age an integral part of modern reality (Barbaccia et al., 2022). United Nations (UN) and World Health Organization (WHO) projections indicate that the number of seniors will double in three decades, reaching 1.5 billion people in 2050 (United Nations, 2020; World Health Organization [WHO], 2020), and people aged 60 and older will make up about 22% of the world’s population (United Nations, 2019). Old age, in addition to its inevitable time dimension, comes with limitations and difficulties in maintaining physical activity that promotes physical and mental health (Viswanathan and Sudarsky, 2012; Chen et al., 2021). These individuals are more prone to falls due to the natural aging process, which negatively affects muscle strength, tissue flexibility, and overall body stability (Moreland et al., 2004; Carapeto and Aguayo-Mazzucato, 2021). Pain and limited joint mobility are the cause of reduced physical fitness, leading to an increased risk of falls and loss of independence (Rubenstein, 2006; Bischoff-Ferrari, 2011; Stevens and Lee, 2018). Activities aimed at strengthening muscles, improving endurance, exercising cognitive function, and reaction speed help prevent falls (Nelson et al., 2007; Zhang et al., 2015; Chantanachai et al., 2021). These elements are an integral part of the recommended holistic approach to maintaining physical and psychosocial fitness in the elderly. Regular balance training in older people contributes significantly to improving stability, reducing the risk of balance loss and falls (Marzetti et al., 2017).

Among the elderly, the limited variety of exercises and their repetition is often the cause of lack of commitment and boredom. Therefore, modern training systems have begun to be introduced to complement conventional work methods and provide the much-desired element of positive and strong motivation (Evans, 1999; Ehrari et al., 2020). The dynamic development of IT/ICT technologies (information technology/information communications technology) and their use also allows the elderly to enter a new digital world – attractive virtual reality – creating an atmosphere of curiosity, even enjoyment, and a significant increase in motivation for regular exercise both in clinical facilities and at home under the supervision of a physiotherapist. Many systems have already been developed to serve these purposes by incorporating virtual reality (VR) into physical activity work. One of these is the ActivLife platform used in our study. It is tailored to prevent falls, especially in people at high risk of falling, providing appropriate and safe support/training and, just as importantly, cognitive support. The system offers a variety of physical exercises, also including a variety of tasks to improve cognitive function. This combination of activities allows us to treat elderly persons precisely holistically, and their health as a complex phenomenon, where physical health is also influenced by emotional wellbeing and cognitive health. The proposed exercises and tasks allow participants/seniors to activate each muscle group, improve joint mobility, and enhance fitness using an attractive form of interactive games, cognitively engaging and creating an atmosphere of fun. However, being in virtual environments (VEs) can be associated with adverse symptoms of cybersickness (Séba et al., 2023; Kourtesis et al., 2024). This is more often experienced by immersive VE participants, and very rarely by non-immersive VR users (Venkatakrishnan et al., 2023; Sokołowska, 2024). Despite these (a) unfavorable effects (Drazich et al., 2023; Dopsaj et al., 2024), but also (b) the lack of standardization of virtual tools/environments (Kourtesis et al., 2021; Porffy et al., 2022; Kim et al., 2023; Holmqvist et al., 2024), (c) researching to prepare recommendations for the use of VR in specific patient populations (Juras et al., 2019; Brassel et al., 2021; Liu et al., 2022; Rodríguez-Almagro et al., 2024), (d) discussing emerging user data protection/privacy issues and ethical dilemmas (Segkouli et al., 2023; Goldstein et al., 2024; Rudschies and Schneider, 2024), researchers and clinicians/physiotherapists highlight the enormous potential of innovative technologies (Jonson et al., 2021; Bateni et al., 2024; Moulaei et al., 2024). Today’s societies are aging at a very rapid pace, which necessitates measures to support the elderly in preventing falls and their serious consequences, including exercising cognitive function in progressive senile dementia (Barbaccia et al., 2022; Yang J. G. et al., 2022; Buele et al., 2023; Ren et al., 2024; Siette et al., 2024; Tortora et al., 2024; Wilf et al., 2024) – these actions are the future of modern clinical practice in (neuro)geriatrics.

Current research demonstrates that the application of virtual environments is effective in supporting such important balance training in older adults. Various virtual protocols are being proposed and tested, showing comparable or even greater benefits of VR training compared to traditional physical training. An important thread related to the development of novel technologies is the computational approach for evaluating the usability and effectiveness of various systems offered via VR (Cavedoni et al., 2020; Yang A. H. X. et al., 2022; Nieto et al., 2024; Veneziani et al., 2024; Wen et al., 2024). In clinical applications, machine learning algorithms are proving to be very useful (Bao et al., 2019; Eichler et al., 2022). Machine learning (ML) (a) is a type of artificial intelligence (AI) focused on building computer systems that learn from data, (b) is a powerful tool for solving problems, streamlining various complex operations, and automating tasks, and (c) has broad applications in many areas, for example, science, engineering, industry, economics, databases, healthcare, and medicine (Michalski et al., 2013; Alpaydin, 2016; Zhu et al., 2020; Sarker, 2021; Singh et al., 2021; Barton et al., 2024; Haimovich et al., 2024; Khalid et al., 2024). ML offers a wide range of techniques, such as decision trees, rule induction, neural networks, support vector machines (SVMs), clustering and classification methods, association rules, feature selection procedures, visualization, graphical models, or genetic algorithms; which are many more complex and use techniques well beyond traditional statistical techniques [i.e., hypothesis testing, experimental design, ANOVA, linear/logistic regression, generalized linear model (GLM), or principal component analysis (PCA)] (Mitchel, 1997; Ben-David and Shalev-Shwartz, 2014; Marsland, 2015; Arnold et al., 2019; Bradley and Trevor, 2021).

Moreover, in the context of the presented significant and already global problem of aging and the consequences of falls among seniors, methods for assessing balance and developing effective methods of maintaining it in various situations are important (Silva et al., 2017; Lo et al., 2019; Roshdibenam et al., 2021; Albites-Sanabria et al., 2024; Song et al., 2024). Posturographic techniques are often used for this purpose (Phu et al., 2019; Błaszczyk and Beck, 2023; Liang et al., 2024; Pennone et al., 2024), also in our research relies on posturography (Sokołowska et al., 2015, 2018; Sadura-Sieklucka et al., 2023).

The study aimed to evaluate and compare the effectiveness of training using a non-immersive virtual environment, such as balance-strength training, but additionally balance-cognitive training, in preventing balance loss and fall risk in older adults. The effect analysis proposed an approach using ML methods, which are increasingly used in medical applications of new technologies due to their high effectiveness.

## 2 Materials and methods

### 2.1 Participants

The study included 36 women aged 62–87 who were patients of the National Institute of Geriatrics, Rheumatology and Rehabilitation in Warsaw. Before starting the training program, they were randomly assigned to two groups, with only the experimenter having knowledge of the type of group. Eighteen of them took part in balance-strength training (VR BST), and 18 in balance-cognitive training (VR BCT) using the ActivLife virtual platform (Alreh Medical, Inc., Poland). The mean age of the participants was 73.8 ± 6.2 years, height 1.61 ± 0.05 m, and weight 68.9 ± 10.6 kg. In the group with balance-strength training, the age was 73.9 ± 6.1 years, height 1.62 ± 0.04 m and weight 69.8 ± 10.2 kg. In the group with balance-cognition, age was 73.7 ± 6.6 years, height 1.60 ± 0.05 m and weight 67.9 ± 11.2 kg. Inclusion criteria for the study were age over 60 and the ability to move independently (Stuckenschneider et al., 2023). The subjects had no history of falls. Exclusion criteria included persistent or transient disturbance of consciousness, moderate and severe dementia, advanced hearing loss and visual impairment, or a serious condition of the subjects due to serious illness or major life events. The study was conducted in accordance with the Declaration of Helsinki, and approved by the Ethics Committee of the National Institute of Geriatrics, Rheumatology and Rehabilitation in Warsaw for research involving humans, no. KBT-2/2/2023. All subjects gave written consent to participate in the study.

### 2.2 Clinical balance tests

#### 2.2.1 *TUG* and *FTSS* tests

The study used standardized tests and examined static and dynamic balance, and lower limb strength to evaluate the effectiveness of training by the Timed Up and Go (*TUG*) test and the five-time sit-to-stand (*FTSS*) test (Beck Jepsen et al., 2022; Poncumhak et al., 2023). The *TUG* test involves getting up from a standard chair, walking a distance of 3 m, turning around, returning to the chair, and sitting. The *FTSS* test involves sitting and standing up straight 5 times as quickly as possible, with no breaks in between, in addition to arms crossed over the chest (Buatois et al., 2010). For these tests, lower times mean better scores and the risk of recurrent falls corresponds to times >15 s. The study assessed participants for fall risk based on the *TUG* test with a cutoff point of 10 s; above this value, the test subjects were classified as having an increased risk of falling (Podsiadlo and Richardson, 1991). The tests were always conducted by the same experimenter using a stopwatch.

#### 2.2.2 Romberg tests with eyes open and closed

Static posturography can be used as an objective tool to complement clinical balance tests to assess and control balance (Błaszczyk and Beck, 2023). The posturography exam was performed with a 30-s Romberg test with eyes open (EO) and eyes closed (EC), using a FreeMed Maxi posturography mat (Koordynacja, Inc., Poland) by the same experimenter. The Romberg test is performed as follows: (a) the subject stands with feet together, eyes open and hands spread to the sides, and then (b) the subject closes the eyes while the examiner observes the person for 30 s. An important feature of this test is that the person becomes more unsteady with eyes closed. The FreeMed Maxi consists of a plate equipped with sensors that measure the distribution of pressure force and changes in the projection of the center of pressure (COP) on the support surface. The body sways can be translated into COP values in the medial-lateral and anterior-posterior directions, and the trajectory (stabilogram/posturogram) represents the displacement of COP in these two directions, projection onto the *X* and *Y* axis, respectively, during the posturographic exam (Sokołowska et al., 2018; Sadura-Sieklucka et al., 2023). The following COP trajectory (posturogram) parameters were recorded and calculated: total COP path length (*TL*), average COP velocity (*V*), COP ellipse area (predictive ellipse area with 95% COP values) (*EA*), and two lengths of directional components of sways in left-to-right frontal and forward-backward plane movements (*LRL* and *FBL*, respectively).

#### 2.2.3 Course of the study

**FIGURE 1:**
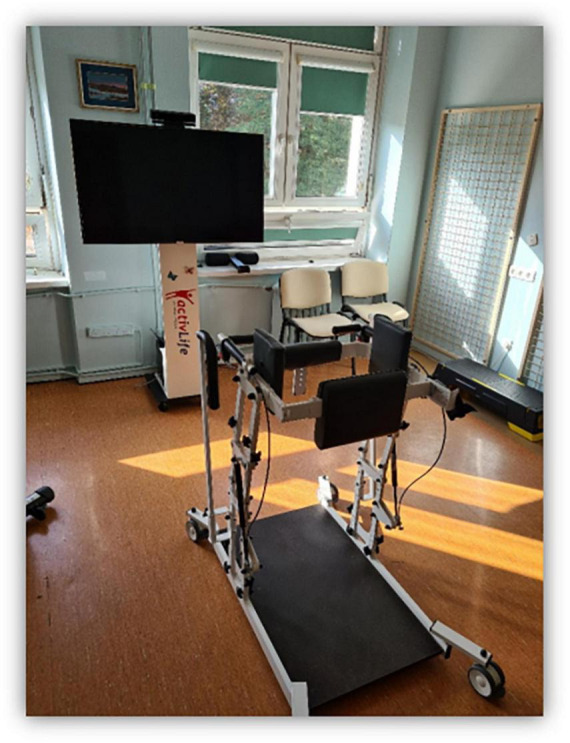
View of ActiveLive set with a GymUp technology corset, screen, and Kinect 3D camera. (The material comes from our NIGRiR repository).

In the present study, clinical tests (*TUG*, *FTSS*, and Romberg with EO and EC) were conducted twice, i.e., before participation in a 10-day training program using a non-immersive virtual environment and immediately after its completion. The training sessions used the ActivLife platform, which offers various virtual physical and cognitive exercises. The ActivLife system consists of a parapodium-based frame equipped with a corset with a seat and an integrated GymUp system (to assist in getting up from a squat), a large screen and a Kinect 3D camera, as shown in Figure 1.

The outcome measures were as follows: (a) the primary outcome was functional balance assessed by *TUG* and *FTSS* scores and (b) the secondary outcomes were static balance parameters assessed by the posturographic trajectory pattern in the Romberg test with EO and EC.

### 2.3 Virtual reality-based training sessions

**FIGURE 2:**
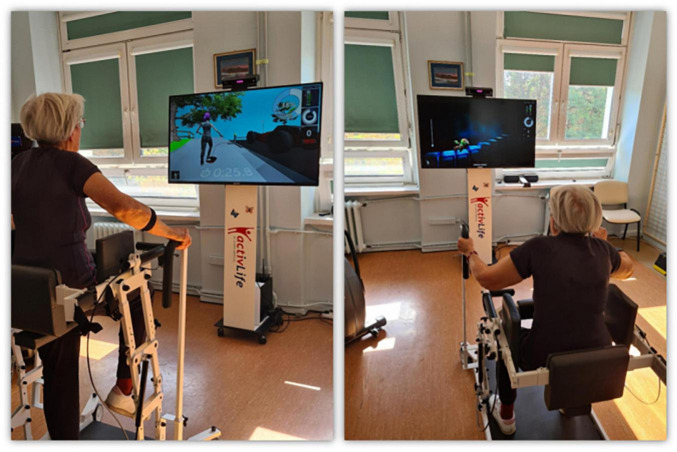
Illustration of a virtual balance-strength training session. The **left panel** presents the “Right Leg Push-up” exercise. The starting position is a standing position with hands resting freely on the handle. The exercise aims to inflate the car tire as quickly as possible by bending the knee and hip joints. Increasing the difficulty of an exercise increases its duration. The **right panel** presents the “Stairs” exercise. The starting position is standing and the hands hold specially adapted handles. The exercise aims to climb the appropriate number of stairs by performing a full squat and returning to the starting position. The difficulty level of the exercise concerns increasing its frequency, i.e., shortening the interval between steps and increasing the number of steps. (The material comes from our NIGRiR repository).

**FIGURE 3:**
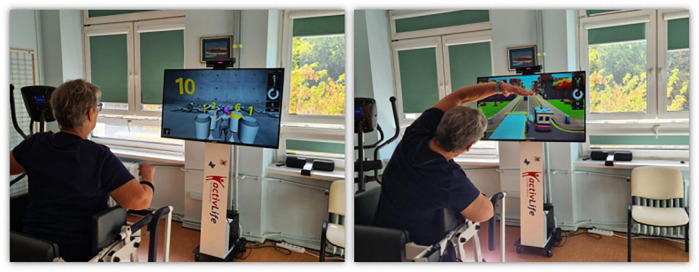
Illustration of a virtual balance-cognitive training session. The **left panel** shows the “Counting” exercise. The starting position is standing with arms along the body. The exercise aims to reflect the number on the side by adding bags. The bags are added up by touching and highlighting them. The difficulty level of the task concerns increasing the range of numbers. The **right panel** illustrates the “Ambulance” exercise. The starting position is with arms resting freely, and the exercise is aimed at avoiding cars in front of the ambulance by side-bending the torso. Increasing the difficulty level of the task is to accelerate the ambulance and reduce the distance between the cars. (The material comes from our NIGRiR repository).

The subjects participated in a 10-day program that included conventional physical training and exercises in a VE created by the ActivLife platform. A single session lasted about 30 min. Each group included 15 min of exercises on a bicycle ergometer, followed by 15 min of exercises aimed at improving lower limb strength, trunk stabilization, and balance in balance-strength training in group 1 (Figure 2), corresponding strength and cognitive exercises in balance-cognitive training in group 2 (Figure 3). The progression of exercises in subsequent sessions of the program consisted of increasing their intensity, the speed at which each exercise/task was performed, and increasing the number of repetitions. One of the common and effective machine learning algorithms, based on the *k*-nearest neighbors (*k*-NN) learning rule, was used to evaluate the effects of the training program.

### 2.4 Data analysis

#### 2.4.1 Machine learning and *k*-NN algorithm

Machine learning is often classified by the way an algorithm learns to become more accurate in its predictions. There are four basic types of machine learning, such as supervised, unsupervised, semi-supervised, and reinforcement learning (Bishop, 2006; Kroese et al., 2019; Alnuaimi and Albaldawi, 2024a,b). In supervised learning, data scientists provide algorithms with labeled training data and define variables. Both the input and output of the algorithm are specified in supervised learning. There are several supervised learning algorithms. Some of the commonly used algorithms of supervised learning are *k*-NN, naive Bayes classifiers, decision trees, logistic regression, and SVMs (Hastie et al., 2009; Pandey et al., 2019).

**FIGURE 4:**
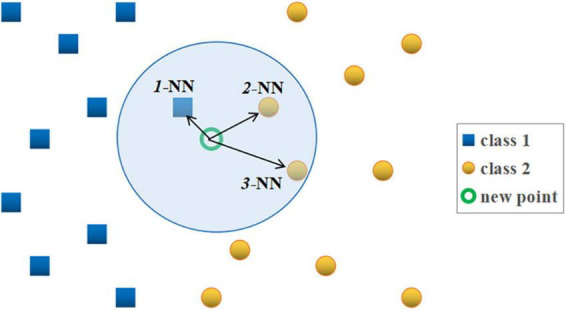
Illustration of the standard *k*-NN rule. “Blue cube” – points belonging to class 1 and “yellow ball” – points belonging to class 2. The symbols *1*-NN, *2*-NN, and *3*-NN stand for the first, second and third nearest neighbor of the new classified point (marked with a “green circle”), respectively. According to the *3*-NN rule, the new point “circle” is assigned to class 2, since two, out of its three nearest neighbors are from class 2 (“cube” – points from class 1, “ball” – points belonging to class 2).

**FIGURE 5:**
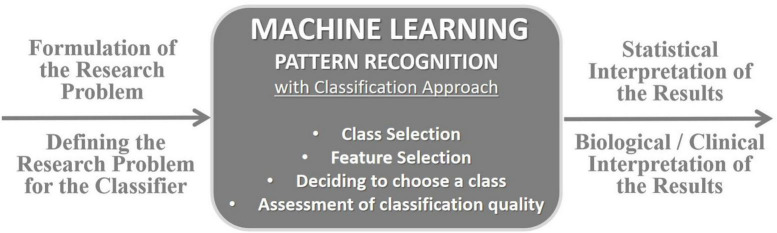
Model of analysis approach based on machine learning with supervised classification employed in our research, e.g., in Sokolowska and Jozwik (2007), Maciejewska et al. (2008), Sokołowska et al. (2008, 2018), Jóźwik et al. (2011), and Sokołowska and Sokołowska (2019).

The *k*-NN is a non-parametric supervised learning classifier that uses proximity to make classifications or predictions about the grouping of an individual data point (an object), which is simple to implement, performs well in practice and can be easily extended to new data. In classification problems, the class label is assigned based on the majority vote – i.e., the label that is most frequently represented around a given data point is used, as shown in Figure 4 (Devijver and Kittler, 1982; Duda et al., 2000; Jain et al., 2000). The classifier quality criterion, depending on the number of the *k*-nearest neighbors, is called the error or misclassification rate (*E*_*r*_), defined as *E_*r*_* = *m*/*n*, where *m* is the number of misclassified objects, and *n* is the total number of the objects in the reference set. The *E*_*r*_ is calculated for all possible values of *k* using the *leave-one-out* method (Duda et al., 2000). A value of *k* should be determined in such a way that offers the smallest probability of misclassification. The lower the *E*_*r*_, the easier it is to differentiate classes. The *k*-NN makes no assumptions about the data, which means it can be used to solve a wide variety of problems (Figure 5).

In agreement with the aim of the study, in the analysis using the *k*-NN classifier, two classes were defined corresponding to the two test groups: class 1 was the balance-strength training group (VR BST), and class 2 was the balance-cognitive one (VR BCT), which additionally takes into account an important cognitive component in balance training. Both primary clinical and secondary posturographic outcomes were analyzed. Based on the posturographic measurements, the effects before and after 10 VR sessions were analyzed: feature *1* – total length of the posturographic trajectory (*TL*), feature *2* – velocity of body swings (*V*), feature *3* – ellipse area of the trajectory (*EA*), feature *4* – length of left-to-right frontal plane motions, as X displacement (*LRL*), and feature *5* – length of the forward-backward length of movements in the sagittal plane, as Y displacement (*FBL*). The analysis was performed for the Romberg test with EO and EC, additionally without and with the feature selection procedure (to indicate the features most strongly related to the classes). The redundant features make the classification more complex and spoil its performance. For this reason, the feature selection is recommended. We should select the feature set, out of all available features, which offers the minimum value of the error rate for the optimum *k-*NN rule. A similar analytical approach was used in our research using a posturography platform, e.g., in Sokołowska et al. (2015, 2018), Sadura-Sieklucka et al. (2023), among others. Figures 4, 5 illustrate the operation of the *k*-nearest neighbors rule and the classification model approach adopted in our research.

#### 2.4.2 Statistical analysis

The statistical package STATISTICA (StatSoft Poland Inc., version 12) was used, as well as computational algorithms based on the standard *k*-NN learning rule (for Euclidean distance) and the *leave-one-out* method for assessing the quality of classification, and also a feature selection procedure. These algorithms are presented and described in Maciejewska et al. (2008), Jóźwik et al. (2011), Sokołowska et al. (2018), among others. Additionally, the analysis used (a) a two-factor repeated measures ANOVA with the Tukey HSD test as a *post-hoc* test (Table 1), and (b) a χ^2^ test with Yates’s correction or Fisher’s exact test (Table 3) to assess the level of statistical significance of the obtained results (for two VR programs before and after VR training). A Shapiro–Wilk test was performed to evaluate the normality of parameters in each group. The value of statistical significance was *p* < 0.05.

**TABLE 1:** Meanand standard deviation of parameters of clinical balance tests before and after training sessions using VR for balance-strength training (BST) and balance-cognitive training (BCT).

Parameters in various conditions		All participants (*N* = 36)	VR BST (*N* = 18)	VR BCT (*N* = 18)
**Classical clinical tests**				
*TUG* (s)	Before	9.1 ± 2.2	9.1 ± 2.1	9.0 ± 2.4
	After	8.2 ± 1.5**	8.1 ± 1.2**	8.4 ± 1.7
*FTSS* (s)	Before	15.6 ± 4.1	15.1 ± 3.4	16.1 ± 4.8
	After	13.5 ± 3.1***	13.1 ± 2.1*	13.9 ± 3.9**
**Romberg test with EO**				
*TL* (mm)	Before	254 ± 67	254 ± 70	255 ± 67
	After	241 ± 63	240 ± 61	242 ± 66
*V* (mm/s)	Before	8.7 ± 2.3	8.7 ± 2.3	8.7 ± 2.3
	After	8.2 ± 2.1	8.2 ± 2.1	8.2 ± 2.2
*EA* (mm^2^)	Before	102 ± 127	86 ± 94	119 ± 155
	After	74 ± 97	42 ± 32	107 ± 127^#^
*LRL* (mm)	Before	6.0 ± 3.9	4.8 ± 3.3	7.2 ± 4.2
	After	5.2 ± 4.5	5.9 ± 5.1	4.5 ± 3.8
*FBL* (mm)	Before	13.1 ± 8.9	12.2 ± 8.1	14.0 ± 9.8
	After	12.5 ± 8.2	11.9 ± 6.9	13.2 ± 9.5
**Romberg test with EC**				
*TL* (mm)	Before	285 ± 85	292 ± 95	277 ± 76
	After	269 ± 61	272 ± 61	266 ± 58
*V* (mm/s)	Before	9.7 ± 2.8	9.9 ± 3.2	9.4 ± 2.5
	After	9.0 ± 2.4	8.7 ± 2.7	9.2 ± 2.1
*EA* (mm^2^)	Before	95 ± 94	91 ± 87	100 ± 103
	After	52 ± 45*	49 ± 45	54 ± 46
*LRL* (mm)	Before	6.1 ± 3.4	5.8 ± 3.4	6.4 ± 3.4
	After	5.8 ± 5.0	6.7 ± 4.4	4.9 ± 5.6
*FBL* (mm)	Before	12.1 ± 6.3	12.9 ± 5.2	11.3 ± 7.3
	After	11.9 ± 7.6	11.2 ± 6.7	12.5 ± 8.6

*TUG*, Timed Up and Go test; *FTSS*, five-time sit-to-stand test; *TL*, total length of the trajectory; *V*, velocity of body swings; *EA* ellipse area of the trajectory; *LRL*, length of left-to-right frontal plane movements; *FBL*, forward-backward length of movements in the sagittal plane; EO, eyes open; EC, eyes closed. Symbols used for two-factor repeated measures ANOVA:

(a) *p < 0.05,

**p < 0.01,

***p < 0.001 for comparisons before and after VR training in VR BST and VR BCT; and

(b) ^#^*p* < 0.05 between VR BST and VR BCT.

## 3 Results

The results obtained by the participants in the *TUG* and *FTSS* tests before and after the 10-day training program in the non-immersive virtual environment are shown in Table 1. The results (of all participants) demonstrate statistically significant favorable changes in both tests between pre- to post-training values, from 9.1 ± 2.2 and 15.6 ± 4.1 to 8.2 ± 1.5 and 13.5 ± 3.1 s, respectively. Moreover, among the subjects, *TUG* test values >10 s were found in 13 subjects (36%) before training (7 in group 1 and 6 in group 2), and after training in only two of them (6%) (one each in both groups). These changes indicate a statistically significant reduction in the risk of falls in individuals from both groups (*p* = 0.0015). However, the observed differences in these clinical tests between the two training groups are small and do not reach statistical significance. This indicates similarly favorable effects achieved by exercise participants in groups 1 and 2, i.e., equally effective fall prevention and balance maintenance. Table 1 also presents the results of the Romberg test with eyes open and closed for posturographic trajectory parameters, and showed non-significant differences between both virtual training sessions, except the *EA* parameter during EO, whose value significantly decreases after VR BST compared to VR BCT, i.e., (a) before 86 ± 94 versus 119 ± 155 mm^2^ (*p* = 0.453), and (b) after 42 ± 32 versus 107 ± 127 mm^2^ (*p* = 0.043), respectively. In turn, the same parameter in the EC test decreased statistically significantly after the virtual program (of all participants), i.e., from 95 ± 94 (value before) to 52 ± 45 mm^2^ (value after) (*p* = 0.014), but this change does not reach statistical significance in either VR BST (from 91 ± 87 to 49 ± 45 mm^2^, *p* = 0.079) or VR BCT (from 100 ± 103 to 54 ± 46 mm^2^, *p* = 0.093) independently.

An analytical model based on machine learning algorithms with supervised learning, i.e., a *k*-NN classifier with known class membership, was proposed and used to evaluate the effects of the VR program. The estimation was based primarily on class recognition of the posturographic trajectory pattern. Table 2 shows the results of distinguishing the training program into two classes, class 1 – virtual balance-strength training (VR BST) and class 2 – virtual balance-cognitive training (VR BCT) for each feature independently, i.e., recorded/calculated posturographic parameters. Table 3 shows the results of analyzing a set of all these features together before and after feature selection, indicating the features that best differentiate the classes (feature selection reduced the error rate). Table 4 summarizes the results of the *k*-NN-based classification (after feature selection) in the form of confusion and confidence matrices.

**TABLE 2:** Results of the *k*-NN analysis for single posturographic features/parameters in distinguishing between two classes, corresponding to VR BST and VR BCT (for EO and EC tests).

Features of posturographic trajectory	EO test		EC test	
	*k*-NN	*E*_*r*_	*k*-NN	*E*_*r*_
1 – *TL*	*K* = 5	0.417	*K* = 7	0.333
2 – *V*	*K* = 5	0.389	*K* = 1	0.361
3 – *EA*	*K* = 25	0.333	*K* = 15	0.417
4 – *LRL*	*K* = 28	0.417	*K* = 2	0.278
5 – *FBL*	*K* = 4	0.444	*K* = 19	0.361

The optimal number of *k*-nearest neighbors and *E*_*r*_ error rate are given. *TL*, total length of trajectory; *V*, velocity of body swings; *EA*, ellipse area of trajectory; *LRL*, length of left-to-right frontal plane movements; *FBL*, forward-backward length of movements in the sagittal plane; EO, eyes open; EC, eyes closed.

**TABLE 3:** Results of *k*-NN analysis for the full set of features/posturographic parameters in distinguishing two classes, corresponding to VR BST and VR BCT, without and with feature selection.

Statistical parame-ters	EO test		EC test	
	Without selection	With feature selection	Without selection	With selection
*K*-NN	*K* = 4	*K* = 5	*K* = 13	*K* = 2
*E*_r_	0.444	0.278	0.389	0.278
Set of features	{1,2,3,4,5}	{1,2}	{1,2,3,4,5}	{4}
*p*(χ^2^)	0.345	0.019	0.264	0.001

The results are presented for EO and EC tests. The optimal number of *k*-nearest neighbors, *E*_*r*_ error rate and *p*(χ^2^) are given.

**TABLE 4:** Confusion (panel A) and confidence (panel B) matrices for *a priori* and *a posteriori* probabilities for selected posturographic features for EO ({*TL*,*V*}) and EC ({*LRL*}) tests.

A. Probabilities that a case from the class *i* (row) will be assigned to the class *j* (column)			B. Probabilities that a case assigned to the class *i* (row) comes in fact from the class *j* (column)		
**EO test**					
True class	Assigned class		Assigned class	True class	
	1	2		1	2
1	0.667	0.333	1	0.750	0.250
2	0.222	0.778	2	0.300	0.700
**EC test**					
True class	Assigned class		Assigned class	True class	
	1	2		1	2
1	1.000	0.000	1	0.643	0.357
2	0.556	0.444	2	0.000	1.000

The accuracy of the *k*-NN classifier was 0.722 (*E*_*r*_ = 0.278) for distinguishing between VR BST (class 1) and VR BCT (class 2).

As shown in Table 2, in the posturography exam with EO, lower misclassification errors (*E_*r*_)* are offered by single features no. *2* and *3*, relating the parameters of *V* and *EA*. However, in the EC test, lower *E*_*r*_ values (compared to the EO test) were observed for other features no. *1* and *4*, corresponding to *TL* and *LRL*. Additional differentiation (not shown in the table) of the two virtual training according to the *TUG* test yields an *E*_*r*_ equal to 0.333 (*3*-NN), and *E_*r*_* = 0.361 (*4*-NN) according to the *FTSS* test. The set of these clinical tests together {*TUG,FTSS*} gives an *E*_*r*_ equal to 0.361 (*6*-NN). Therefore, it is more effective to distinguish between classes according to the *TUG* feature, correctly identifying both classes in 67% (*p* = 0.046). As Table 3 shows, considering all features (all posturographic parameters), i.e., the set of features {*1,2,3,4,5*}, yields a large *E*_*r*_ (0.444 in the EO test and a slightly lower 0.389 in the EC one). This situation is improved by the feature selection, which also indicates important parameters in recognizing training effects (classes considered). After feature selection, a set of two features {*1,2*} is significant in the EO test, i.e., *TL* and *V* (*E_*r*_* = 0.278). In the EC test, a single feature no. *4*, i.e., *LRL*, at the same *E*_*r*_ differentiated the two classes. As shown in Table 4, the confusion matrix for selected features (i.e., panel A for *a priori* probabilities), (a) can be seen that (a) the fraction of correct decision was obtained for class 1, 0.667 for the EO test and 1.000 for the EC test, (b) for class 2 was 0.778 for EO and only 0.444 for EC. In addition, panel B also shows a confidence matrix (for *a posteriori* probabilities), i.e., the probabilities that the case assigned to a row class comes, in fact, from a column class. As can be seen, (a) in the case of EO, slightly more correct assignment decisions apply to class 1 compared to class 2 (the probabilities of correct decisions are 0.750 and 0.700, respectively, (b) while for EC, class 2 was precisely assigned (1.000), class 1 was correctly assigned with a probability of 0.643. The accuracy of the *k*-NN classifier in distinguishing between VR BST and VR BCT was 0.722 (*E_*r*_* = 0.278).

As expected, while differentiating the effects of virtual sessions, the inclusion of two additional features in the considered set of posturographic features (related to the *TUG* and *FTSS* tests) does not improve the results based on the posturographic pattern. For such an extended set of features, *E_*r*_* = 0.472 (*13*-NN) was obtained in the EO test and *E_*r*_* = 0.472 (*8*-NN) in the EC test, and after feature selection, those lower values of 0.278 (*9*-NN) and 0.333 (*1*-NN) were observed, respectively.

All participants reported that the exercises were attractive and engaging. None of them experienced adverse virtual symptoms during the VR program.

## 4 Discussion

Our research deals with a computational approach based on machine learning algorithms in the very timely topic of using cutting-edge VR-based technologies to prevent falls, a very serious problem in the elderly. The study involved 36 women aged 62–87. They participated in two different training sessions with the same non-immersive VR platform designed for fall prevention training, i.e., VR BST (*n* = 18) and VR BCT (*n* = 18). The main difference between the VR sessions was the inclusion of cognitive tasks in the VR BCT in addition to the required strength exercises. The results of classical clinical tests such as the *TUG* and *FTSS* showed similarly beneficial effects of both virtual training. Clinical Romberg tests along with posturographic trajectory pattern analysis were also used to evaluate the effects of virtual training. The analytical model was based on both classical statistics and the ML supervised learning algorithm with the *k*-NN classifier. Distinguishing the effects of VR BST and VR BCT was a difficult task for the classifier, which it handled well, achieving an accuracy of 0.722 (72%) based on a set of posturographic features/parameters along with the use of a feature selection procedure. In the classical statistical analysis, the vast majority of comparisons did not reach statistical significance.

This was due, among other things, to the fact that there were common elements of exercising motor functions (in terms of balance, coordination, and reaction speed) to maintain a stable posture in both proposed training sessions. The training programs were designed to effectively support individuals at (high) risk of falling (in the case of the presented study of seniors). It is worth emphasizing that posturography is one of the objective methods/techniques of evaluating the human balance system both in healthy and unhealthy individuals (Lipowicz et al., 2023; Oczadło et al., 2023). In particular, static posturography is a simple non-invasive technique commonly used in modern laboratories and clinics to quantify the adaptive mechanisms of the central nervous system involved in postural and balance control (Błaszczyk and Beck, 2023). The main value of a posturography exam is the objective information it provides, making it possible to evaluate: (a) different sensory systems involved in balance (vestibular, visual, and somatosensory), (b) changes of automatic and voluntary motor responses, (c) postural strategies, (d) deviations from the center of gravity, and (e) changes of limits of stability (Schubert et al., 2012a,b; Błaszczyk and Beck, 2023; Oczadło et al., 2023). Posturography is, therefore, an adequate tool for conducting our research with seniors, both now and in the future [including dynamic or virtual posturography (Sokołowska et al., 2020, 2022)]. As expected, the results show that choosing a posturographic exam with the Romberg test with EO, that is, under the subjects’ visual control, effective in analyzing body swings in terms of total trajectory length and sway velocity. Other, slightly more favorable balance-stabilizing changes compared to VR BST (group 1) were observed in VR BCT (group 2), with slightly smaller swings and velocities on average. They allowed 78% of participants in this group to be identified (*p* = 0.019). On the other hand, the results of the EC test (in the absence of visual control) indicate another change in the pattern by a slight decrease in the average *LRL* in group 2, while it increases in group 1. This direction of change allows 100% of participants in group 1 to be identified (*p* = 0.0014) and is probably due to the predominance of motor tasks and, consequently, the greater ranges of motion required in balance-strength tasks compared to group 2 in balance-cognitive tasks.

Summarizing the results of our study, we note that both virtual training sessions, according to the results of the clinical *TUG* and *FTSS* tests, are effective in reducing the risk of falls and balance loss in participants. As Bohannon points out (Bohannon, 2006), the *FTSS* test is used to assess the functional capabilities of the elderly population. Both training programs also exercised the lower limbs, resulting in improved scores on the *FTSS* one. In our study, we observed slightly different directions of changes in the pattern describing posturographic trajectories in the two VR training groups based on the posturographic exam with EO and EC in the Romberg test. This indicates that the strategies developed by the participants in the VR BST and the VR BCT were slightly different. It should also be noted that both training programs proved equally effective in achieving the stated goal of the 10-day sessions using the VE, i.e., reducing the risk of falls and postural stabilization. Those who took part in the training did not experience any symptoms related to the use of VR. They also all found the activities attractive and were eager to participate. For example, Liang et al. (2024) also demonstrated the effectiveness of the ML (machine learning) and XAI (explainable artificial intelligence) approach with (static) posturographic parameters to classify fall risk based on *TUG* scores in older adults. Bao et al. (2019), on the other hand, in their study, proposed an automated balance assessment using trunk sway data and ML methods. Their model, evaluated in a *leave-one-participant-out* scheme, achieved a classification accuracy of 82%. The authors suggested that the ML technique could provide accurate assessments during standing balance exercises. Such automated assessments could reduce physiotherapists’ consultation time and increase users’ adherence to recommendations outside the clinic. This research model may also be a good suggestion for balance assessments, such as home programs/exercises for the elderly in fall prevention. In turn, Eichler et al. (2022) also noted the importance of developing automation of fall risk assessment in an efficient and non-invasive way, especially in older adults. This approach could provide a basis for screening individuals for fall risk and determining their need for participation in fall prevention programs. The authors proposed an automated and effective fall risk evaluation system based on a human motion tracking system using a multi-depth camera and the validated Berg Balance Scale (BBS). Trained machine learning classifiers predict the subjects’ 14 scores for BBS tasks. In addition, researchers proposed their efficient BBS system (referred to as E-BBS), which reduces the number of tasks in a conventional BBS test by about 50% (from 14 to just 4–6 tasks) while maintaining 97% accuracy. The authors concluded that their ML-based approach enables the effective diagnosis/recognition of people at risk of falling in a way that does not require significant time or resources from the medical community. The researchers emphasize that the technology and ML algorithms can be implemented in other sets of clinical tests and evaluations. An example of such a universal approach using ML methods can be seen in our current (and previous) research. Interesting results are presented by Yousefi Babadi and Daneshmandi (2021), who analyzed the effect of VR compared to conventional balance training on the balance of elderly people. The researchers showed that after the intervention/training, there were significant improvements in both groups (*p* < 0.05), and the beneficial changes were similar (*p* > 0.05). The authors concluded that both VR and conventional balance training methods are equally effective. They also suggested that VR training programs could be used as a new home training method to improve the balance of the elderly. The researchers also pointed out important facts such as that (a) VR training is a fun way to improve physical activity for the older participants, and (b) VR is a great way to simulate movement and transfer it to real-world tasks. A study by Lima Rebêlo et al. (2021) found that immersive VR was effective in rehabilitating older adults with balance disorders and fall risk, and similarly, VR training (VRT) was no better than conventional physiotherapy. Moreover, improvements in functional balance after 2 months were maintained after both trainings. In contrast, Liu et al. (2022) quantitatively analyzed the effects of VRT on functional mobility and balance in healthy older adults in their systematic review and meta-analysis, conducted in 15 studies on a total of 704 participants. The analysis showed that compared to traditional physical therapy (TPT), VRT significantly improved *TUG*. The researchers indicated that VRT can be more effective than TPT in improving functional mobility and balance in the study population of healthy seniors. Similarly, Rodríguez-Almagro et al. (2024) reviewed recent evidence on the effectiveness of VR in improving balance and gait among healthy older adults compared to other therapies. The researchers concluded that VR therapy was more effective than minimal intervention or usual care in improving static balance, dynamic balance, and gait in healthy seniors. The authors indicated that VR therapy gives even better results compared to conventional balance training and exercise in improving balance and gait in this senior population. The authors also confirmed the effectiveness of both virtual and conventional methods. Interesting approaches combining both forms of training are also being used. An example is the study by Sadeghi et al. (2021), which evaluated the effects of 8-week conventional balance training, virtual training, and combined exercise (MIX) in older men. The researchers demonstrated that MIX induced the greatest improvements in lower limb muscle strength, balance and functional mobility of all these workouts, and recommended that MIX is an effective method for reducing the risk of falls among older adults.

As emphasized in the Introduction, it is VEs that offer new, highly attractive, interesting, engaging, and motivating conditions (both in the clinic and at home) designed not only for short-term exercise (e.g., our study), but also provide the opportunity to exercise for longer periods, without fatigue or boredom, depending on the needs and abilities of the exercising individuals. Our research combines conventional training with a virtual environment, thus leveraging the advantages of each for the greater benefit of program participants (e.g., increasing the appeal, engagement, and direct impact of the participant on the training through an interactive game format). Since we use novel VEs in our research, it is worth noting that sometimes users in virtual worlds may experience adverse symptoms due to cybersickness (Lima Rebêlo et al., 2021; Sokołowska, 2023, 2024; Kourtesis et al., 2024). Cybersickness is accompanied by a mix of unfavorable symptoms such as headache, nausea, dizziness, fatigue, oculomotor, and postural disturbances (Kourtesis et al., 2023; Venkatakrishnan et al., 2023). An interesting overview of research on this problem using the machine learning approach was presented by Yang A. H. X. et al. (2022). ML can be used to detect them and is a step toward overcoming these adverse limitations of new technologies. The researchers concluded that while various models for detecting cybersickness have been developed, there is no model to predict early adverse events in VEs. Future, more accurate and effective ML approaches will undoubtedly be inspired by current knowledge of how the brain works, as well as the new brain/organism models/approaches being developed and linking them to these bio-digital frontiers (Benelli et al., 2023; Daşdemir, 2023; Yang et al., 2023). These and similar studies (Chang et al., 2023; Souchet et al., 2023) indicate, as does our research, that we are still at the beginning of understanding the potentially beneficial and detrimental effects of digital worlds on their users, especially older adults (Drazich et al., 2023; Séba et al., 2023).

It should also be noted that, as outlined above, ML techniques have enormous potential, particularly in computational neuroscience. These methods have several key advantages over traditional statistical techniques, including learning from data and making predictions based on patterns and relationships present/hidden in the data (Cristancho Cuervo et al., 2022). Consequently, ML methods in neuroscience and contemporary medical practice provide very effective support for accurate diagnosis (Rosenfelder et al., 2023), assessment of beneficial or adverse effects of therapy and rehabilitation (Yang A. H. X. et al., 2022), as well as natural aging using innovative IT/ICT technologies, which can effectively help maintain joy/satisfaction and good quality of life for the elderly (Yousefi Babadi and Daneshmandi, 2021).

### 4.1 Limitation of our study and prospects

A limitation of our study is the small number of participants. In the future, it would be necessary not only to increase the number of subjects but also to include men. The training program included only 10 sessions in the virtual environment, and increasing the number of sessions would enhance the beneficial effects, and the effects would also be worth evaluating in the longer term. A longer training period allows the exerciser to become more familiar with the techniques and develop a habit of regular physical activity, which can result in long-term maintenance of the positive effects of training (Cadore et al., 2013). Sherrington et al.’s (2017) research indicates the beneficial effects on balance and often strength after longer periods of training. Characteristic problems of the senior population are muscle weakness and balance disorders. It would be interesting to be able to continue training not only as presented in a medical facility, but also at home, under the remote supervision of a physical therapist. To put in perspective, virtual environments can simulate real-life situations and challenges related to balance and fall risks in the elderly while providing them with a safe, controlled, and engaging/motivating training environment in which they can practice and effectively improve their balance.

## 5 Conclusion

First, our study demonstrated the effectiveness of the proposed research approach, using a combination of traditional and VR-based balance/cognitive training, dedicated to people at high risk of falls, including seniors. Second, the results showed that the proposed VR training positively improved *TUG* and *FTSS*, i.e., a statistically significant reduction in the risk of falls in the research population of seniors. Third, the analytical approach based on machine learning algorithms is also useful in VE, not only confirming the effectiveness of the proposed virtual training but also indicating a change in the pattern of posturographic trajectory depending on the type of virtual training, VR BST or VR BCT.

## Data availability statement

The raw data supporting the conclusions of this article will be made available by the authors, without undue reservation.

## Ethics statement

The studies involving humans were approved by the Ethics Committee of the National Institute of Geriatrics, Rheumatology and Rehabilitation in Warsaw. The studies were conducted in accordance with the local legislation and institutional requirements. The participants provided their written informed consent to participate in this study.

## Author contributions

BS: Writing –original draft, Writing –review & editing. WŚ: Writing –original draft, Writing –review & editing. ES-B: Writing –original draft, Writing –review & editing. ES: Writing –original draft, Writing –review & editing. TS-S: Writing –original draft, Writing –review & editing.
